# Ionic liquid multistate resistive switching characteristics in two terminal soft and flexible discrete channels for neuromorphic computing

**DOI:** 10.1038/s41378-022-00390-2

**Published:** 2022-05-26

**Authors:** Muhammad Umair Khan, Jungmin Kim, Mahesh Y. Chougale, Chaudhry Muhammad Furqan, Qazi Muhammad Saqib, Rayyan Ali Shaukat, Nobuhiko P. Kobayashi, Baker Mohammad, Jinho Bae, Hoi-Sing Kwok

**Affiliations:** 1 Department of Ocean System Engineering, Jeju National University, 102 Jejudaehakro, Jeju, 63243 Republic of Korea; 2 Department of Electrical Engineering and Computer Science, Khalifa University, Abu Dhabi, 127788 UAE; 3 System on Chip Center, Khalifa University, Abu Dhabi, 127788 UAE; 4 Department of Electronics and Computer Engineering, The Hong Kong University of Science and Technology, Clear Water Bay, Kowloon, Hong Kong; 5 State Key Laboratory on Advanced Displays and Optoelectronics Technologies, The Hong Kong University of Science and Technology, Clear Water Bay, Kowloon, Hong Kong; 6 Baskin School of Engineering, University of California Santa Cruz, 1156 High Street, Santa Cruz, CA 95064 USA

**Keywords:** Chemistry, Electrical and electronic engineering

## Abstract

By exploiting ion transport phenomena in a soft and flexible discrete channel, liquid material conductance can be controlled by using an electrical input signal, which results in analog neuromorphic behavior. This paper proposes an ionic liquid (IL) multistate resistive switching device capable of mimicking synapse analog behavior by using IL BMIM FeCL_4_ and H_2_O into the two ends of a discrete polydimethylsiloxane (PDMS) channel. The spike rate-dependent plasticity (SRDP) and spike-timing-dependent plasticity (STDP) behavior are highly stable by modulating the input signal. Furthermore, the discrete channel device presents highly durable performance under mechanical bending and stretching. Using the obtained parameters from the proposed ionic liquid-based synaptic device, convolutional neural network simulation runs to an image recognition task, reaching an accuracy of 84%. The bending test of a device opens a new gateway for the future of soft and flexible brain-inspired neuromorphic computing systems for various shaped artificial intelligence applications.

## Graphical abstract

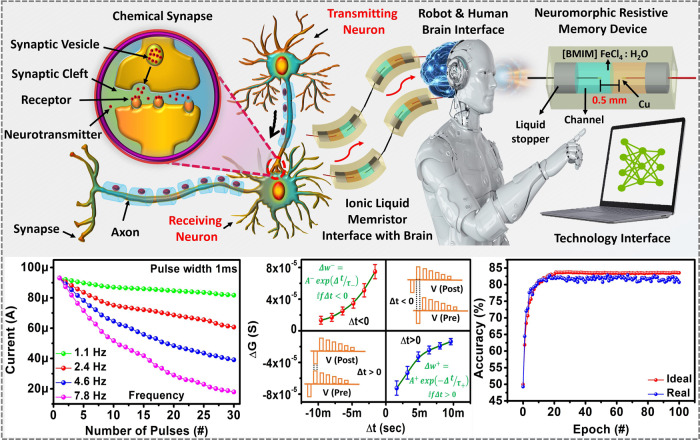

## Introduction

**Fig. 1:**
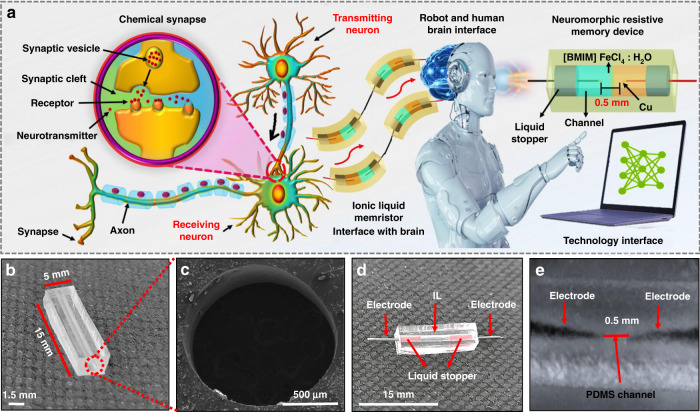
Chemical synapses, device fabrication, and morphological characterizations. **a** Illustrating the synaptic response between the presynaptic neuron and the postsynaptic neuron and ionic memristor interface with future technology. **b** Fabricated PDMS mold with a channel length of 15 mm, **c** and channel width of 1.5 mm. **d** Optical image of the realized device, **e** magnified image of electrode spacing ~0.5 mm

Synapses are the most elegant memory network, in which each neuron is polarized with ions (Ca^+^ or K^+^), which communicates and results in a release of neurotransmitters^1,2^, as shown in Fig. 1a. Similarly, to realize neuromorphic devices, researchers are trying to develop next-generation computing technology by using the nonvolatile conductance property of memristive materials^3^ to emulate synapses^4,5^, as shown in Fig. 1a, which include metal oxides^6–9^, 2D materials^10^, organic materials^11–14^, inorganic materials^15^, hybrid materials^16,17^, and ionic liquids (IL)^18–20^. Hence, liquid materials are receiving more attention due to their high flexibility, high ion conductivity, and easy device fabrication^21^. Ionic liquids and hydrogels are widely used for the fabrication of electronic devices^22–24^. Hydrogels are biocompatible, soft, and have high ion mobility, and their properties can be further improved by adding polyelectrolytes^25–27^. Ionic liquids can be used to fabricate ionic transistors and nonlinear ionic resistors, which can help to control the current–voltage characterizations^28,29^. In such devices, ionic mobility can be represented by the movements of cations and anions^30^. Many researchers are focusing on introducing soft and flexible resistive memory devices using aqueous electrolytes (ionic liquids, hydrogels) as an active material and soft and conductive materials as electrodes^30,31^. Such devices are easy to fabricate with simple fabrication technology, low cost, high flexibility, and good ion mobility with stable performance^32,33^. Liquid materials are beneficial for understanding the device mechanism due to free ion movement (cation and anions), which results in electrode metallization at the anode and cathode^30,32^. To understand the conduction mechanism using ionic liquids, researchers have explored resistive memory devices using different device structures, materials, and aqueous electrodes, such as Ag/AgNO_3_/Probe_Tip_(inert electrode)^31^, Ag/H_2_O/Au^30^, and Au/Trypsin/FTO^33^. Liquid devices can further be used for the biological nervous system to adopt a parallel structure for an energy-efficient computing system^18^.

Signal transmission in biological neurons is defined between presynaptic and postsynaptic neurons^34^, as shown in Fig. 1a. Similarly, multistate resistive switching neuromorphic devices can be used to mimic synaptic function by using the discrete channel, in which the IL will play an important role in the movement of cations and anions^18,21,32^, as shown in Fig. 1a. Specifically, the ability to emulate SRDP and STDP of the soft and flexible discrete channel IL needs to be addressed to perform the electronic synapses^5,18^. However, several critical aspects of liquid-based soft and flexible artificial synapses with memorable conductance tuning under bending and stretching states are required to successfully produce a functioning hardware neural network, which has rarely been reported in discrete channel systems. Many new ionic liquid materials must be introduced to fabricate neuromorphic resistive memory devices to perform electronic synapses. This proposed work introduces a new ionic liquid (IL) BMIM FeCl_4_ and H_2_O into a discrete channel system and thus realizes multistate resistive switching behavior. We then emulate the analog weight change behavior SRDP and STDP of a synapse with our discrete channel memristor and evaluate its performance in a convolutional neural network pattern recognition task based on a system-level simulation. The device presents a highly stable multistate resistive switching behavior in a bending and stretching state. We are confident that soft and flexible devices are excellent candidates for neuromorphic computing in the field of artificial intelligence and brain shape-mimicking robotics, as shown in Fig. 1a.

## Results and discussion

### Physical and electrical characterization

**Fig. 2:**
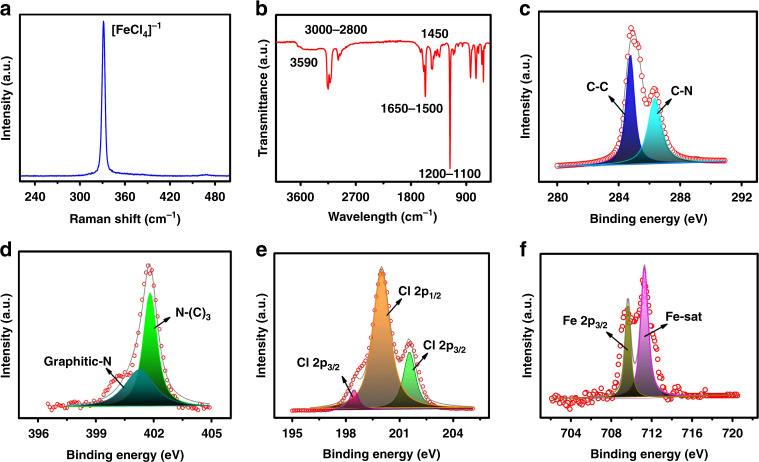
Material characterizations of the BMIM FeC_4_ ionic liquid for the fabrication of the artificial synaptic device. **a** Raman and **b** FTIR of BMIM FeCl_4_. XPS of the **c** carbon, **d** nitrogen, **e** chlorine, and **f** iron regions of BMIM FeCl_4_

The fabricated cylindrical channel is shown in Fig. 1b, and the cross-sectional S.E.M. image at a magnification level of 500 µm is shown in Fig. 1c. The channel filled with ionic liquid (BMIM FeCl_4_ and H_2_O) and electrodes are connected on both ends, as shown in Fig. 1d, and the distance between both electrodes is ~0.5 mm, as shown in Fig. 1e. The chemical characterization was performed by using Raman spectroscopy, FTIR, and XPS. In Raman spectroscopy, vibrational analysis of materials is analyzed at the atomic scale. Every free-standing crystal holds its natural vibration frequency on the lattice and foundation of the material. The Raman spectrum of BMIM FeCl_4_ shows a strong peak at 330.2 cm^−1^, corresponding to the symmetric Fe–Cl stretching vibration of [FeCl_4_]^−^, as shown in Fig. 2a^35^. We performed FT-IR to investigate the functional groups of BMIM FeCl_4_, as shown in Fig. 2b^36^. The hydroxyl group is observed at 3590 cm^−1^ in the FTIR spectra. The –C–H stretching vibrations of the imidazolium cation are observed in the peak range of 3105–3200 cm^−1^. In addition, 3000–2800 cm^−1^ peaks showing the stretching behavior of –C–H, –CH_2,_ and –CH_3_ of the alkyl groups attached to the nitrogen atom in the imidazolium ring of the BMIM FeCl_4_ ionic liquid. The vibrational peaks of the imidazole ring are observed at 1650–1500 cm^−1^ and 1450 cm^−1^. The metal chloride is observed in the transmission bands around 3200–3100, 3000–2800, 1650–1500, 1450, and 1200–1100 cm^−1^. The XPS BMIM FeCl_4_ was measured for the identification of elements and their chemical bonding^37,38^. The C-1s peak was adjusted at 285.00 eV for the calibration of absolute binding energy and represents the deconvoluted spectra of BMIM FeCl_4_, with core levels of Fe-2p, N-1s, Cl-2p, and C-1s from the surface of the sample, as shown in Fig. 2c. The dominant peaks at 284.72 eV and Lorentzian fitted peak at 286.21 eV in the high-resolution spectra of C1s are attributed to the bonding of C–C and C–N, respectively, as shown in Fig. 2c. Analysis of the N-1s core-level line signifies that the Lorentzian fitted peak at 401.23 eV relates to graphitic nitrogen, and other peaks at 401.83 eV show N–C bonding, as shown in Fig. 2d. The high-resolution Cl-2p XPS spectrum demonstrated doublets at 201.8 eV and 200.3 eV associated with the 2p^1/2^ and 2p^3/2^ levels due to spin–orbital coupling, which is a typical indication of the organic C–Cl covalent bond structure. A further subpeak at 198.43 eV demonstrates the coupling of the 2p^3/2^ Cl orbitals, as shown in Fig. 2e. The Fe-2p core-level line has an FWHM of 4.12 eV, and its high-resolution spectra reveal two subpeaks. The dominant peak at 711.3 eV confirms the bonding of Fe-cat, and other peaks at 709.86 eV correspond to the transition of Fe-2p_3/2_ spin orbitals, as shown in Fig. 2f.

### Conduction mechanism

**Fig. 3:**
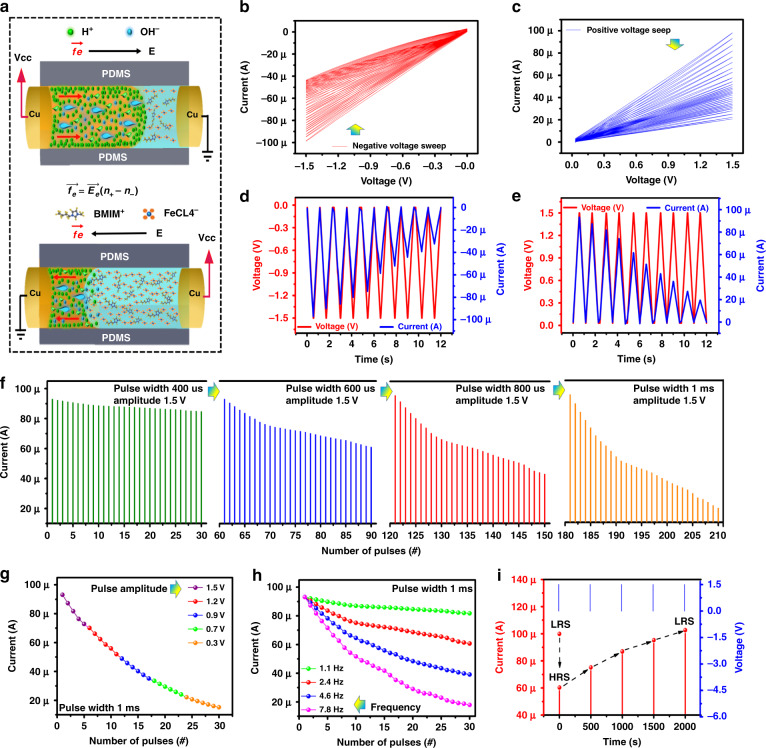
The working mechanism and electrical characterizations of the neuromorphic resistive memory device. **a** Basic mechanism schematic for the electrical manipulation of ion and liquid transport in the discrete channel. The *I*–*V* of **b** negative and **c** positive voltage sweep. The five cycles of triangular voltages with time on **d** negative and **e** positive voltage regions. **f** Plasticity characteristics of the artificial synapse with different pulse widths of 400 μs, 600 μs, 800 μs, and 1 ms. **g** The different pulse amplitude responses at 1.5, 1.2, 0.9, 0.7, and 0.3 V. **h** Frequency response at 1.1, 2.4, 4.6, and 7.8 Hz. **i** The STM retention of the neuromorphic device

The human brain is composed of interconnected neurons, where the presynaptic neuron passes information to the postsynaptic neuron and results in the transmission of neurotransmitters that control synaptic plasticity^18,39,40^. Similarly, the multistate resistive switching characteristics in a two-terminal discrete PDMS cylindrical channel can also mimic biological synaptic plasticity by gradual variation in the resistance state by a repeated pulse sequence to modulate the conduction between the interface of the IL BMIM FeCl_4_ and H_2_O. The composition of the mixture used in the discrete channel in a volume ratio of 1:1 was BMIM FeCl_4_:H_2_O. The ion selectivity (BMIM^+^, FeCl4^−^, H^+^, OH^−^) provides the basic mechanism for discrete channel devices, in which the surface charges on the channel walls will repel ions with the same charges and attract oppositely charged ions^18^. The electroosmotic flow equation, as shown in Fig. 3a, where the counterions attracted to the wall surface charges in H_2_O play the predominant role in the transport rather than the ions in the bulk region of IL BMIM FeCl_4_. The electric body force $\overrightarrow {f_e}$ creates an imbalance between co-ion densities and counterions (*n*_+_−n_−_), which causes a net charge in the presence of the applied electric field $\vec E$ on an interface between the IL BMIM FeCl_4_ and H_2_O, as shown in Fig. 3a. The electroosmotic flow will cause conductance tuning in our discrete channel device to perform neuromorphic computing. The conductance decrease can be observed during electrical body force due to electrode metallization on the cathode and anode, which results in oxidation and reduction. Cu ion movement plays an important role during electroosmotic flow, resulting in ion concentration polarization and electrode metallization. The anode electrode biased with a positive voltage releases Cu^++^ ions in the ionic liquid, and OH^−^ ions move toward the anode and form CuOH_2_. In addition, the cathode was biased with a negative voltage, where Cu^++^ ions in the aqueous electrolyte were reduced to Cu. In this process, ion flow increases in the beginning and saturates beyond the critical voltage point. Due to diffusion of the concentration gradient flux, ionic flow decreases, creating a high resistance state (metallization process). After changing the voltage polarity, Cu^++^ ions will move in the opposite direction, and the conduction filament will break. This process will repeat during each voltage sweep and results in a conductance decrease due to electrode metallization.

### Electrical characterization

The measured multistate resistive switching shown in Fig. 3b, c shows that conductance tuning can be observed with each voltage sweep. Hydration reactions hardly occur between the interface of PDMS and IL BMIM FeCl_4_; for this reason, the surface charges of IL BMIM FeCl_4_ on the channel wall are negligible, and ionic movement is directed in the presence of electrical body force, as shown in Fig. 3a. Hence, we infer that a positive voltage will result in an electrical body force that exists only within the liquid medium and points from the H_2_O side of the device to the IL BMIM FeCl_4_ side, as illustrated in Fig. 3a. This force pushes the H_2_O against the IL BMIM FeCl_4_, as shown in Fig. 3a, and conductance is tuned under continuous voltage sweeps due to the smaller resistivity of the H_2_O compared to IL^18^, as shown in Fig. 3b, c. The above discussion indicates that the discrete channel is equivalent to an interfacial memristor^18^, in which under negative and positive voltage region current decreases with voltage sweeps and conductance of the device decreases Fig. 3b, c. Thus, the electrical characteristics of the soft and flexible discrete channel device can be related to synapses in which the conductance decrease can be related to ion metallization^18^. The measured current under a ten triangular voltage sweep with a period of 1.2 seconds and conducting current decreases with time, as shown in Fig. 3d, e.

### Spike rate-dependent plasticity

The spike rate-dependent plasticity (SRDP) of the multistate resistive switching device in the discrete channel was examined by varying the pulse width, pulse amplitude, and frequencies^39,41^. The synaptic weight can be modulated by the successive stimuli of externally applied pulses of different widths of 400 μs, 600 μs, 800 μs, and 1 ms by keeping a constant pulse amplitude of 1.5 V, as shown in Fig. 3f. The pulse width of 400 μs shows no obvious decrease in the current (93–87 μA). The increase in pulse width up to 1 ms results in a significant change in current from 93 to 19 μA compared to pulse widths of 400, 600, and 800 μs. Different voltage amplitudes of 1.5, 1.2, 0.9, 0.7, and 0.3 V with a pulse width of 1 ms, the current decreases from 94 to 12 µA, as illustrated in Fig. 3g, which corresponds to a lower electrical body force between the interface of IL BMIM FeCl_4_ and H_2_O at a lower voltage compared to a higher voltage. The frequency test is performed using a continuous pulse train on the optimized pulse width of 1 ms and amplitude of 1.5 V, as shown in Fig. 3h. The frequency range of 1.1 Hz shows a very small current variation under a continuous pulse train. On the other hand, by increasing the frequency range up to 7.8 Hz, the current decreasing rate is larger (93 to 18 μA) compared to the previous conditions with lower frequencies (1.1, 2.4, and 4.6 Hz), as shown in Fig. 3h. The performance of neuromorphic resistive memory devices is discussed in Supplementary Table S1.

### Memory retention

This experiment demonstrates the process of memory retention of the multistate resistive switching device. Initially, by applying the input pulses to set the device in high resistance state (HRS). as shown in Fig. 3i. Then, every 500 s, the conductivity was measured by applying 1.5 V with a pulse width of 1 ms. The current increases or recovers by applying the pulses after every 500 s. The neuromorphic device recovers to its initial state of low resistance state (LRS) after 2000 s. The device recovers its state from HRS to LRS, and this phenomenon can be related to the forgetting of human memory^42^. Short-term memory (STM) is an important feature of the human brain, which provides information loss at a particular time^43^. Similarly, it is an important feature to mimic forgetting behavior for electronic synapse devices^44^, as shown in Fig. 3i. The switching transaction by applying input pulses to change the device state from LRS to HRS is similar to the short-term depression (STD)^45,46^.

### Bending and stretching

**Fig. 4:**
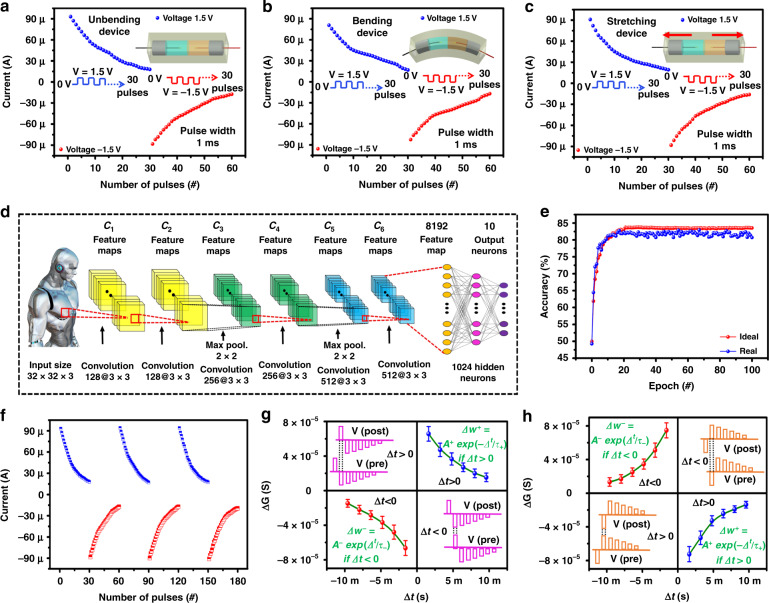
The device flexibility test and implementation of CNN using the weight change behavior of the proposed device. The synaptic plasticity of the **a** unbending, **b** bending, and **c** stretching devices under a continuous pulse train with a pulse of 1 ms. **d** Diagram of neural networks for the recognition of CIFAR-10 data. **e** Simulated accuracy of ideal and real devices. **f** Device endurance performance. The experimental STDP results show **g** antisymmetric Hebbian and **h** antisymmetric anti-Hebbian learning rules

The more sophisticated pulse scheme was implemented to mimic the SRDP, in which the current was obtained by applying 30 consecutive pulses with an amplitude of ±1.5 V and pulse width of 1 ms with a duty cycle of 50%, as shown in Fig. 4a. The gradual variation in the current with pulses is similar to a variable synaptic weight in bio synapses. In the IL BMIM, FeCl_4_ and H_2_O discrete channel synaptic devices behave like brain neuronal activation, in which a similar phenomenon also exists after inverting the polarity of voltage, as shown in Fig. 4a. The scaling possibility of the device depends on the PDMS and ionic liquid (EMIM FeCl_4_), which helps to form the device in any shape by controlling the length, width, thickness, and flexibility. This work provides a demonstration of the adoption of flexible discrete channel resistive memory architectures for neuromorphic computing. This experiment is based on the demonstration of the device’s flexibility to intergrade the discrete channel device in brain shape-mimicking robotics^21^. The bending and stretchability nature of the device was due to the IL BMIM FeCl_4_ and PDMS substrate. In the bending state, the device can be bent from flat down to a 1-m bending curvature, and in the stretching state, the device can be stretched up to a 10% strain limit (further stretching will affect the device performance and stability). The device shows stable analog resistive switching behavior and can be used for neuromorphic computing in soft robotics in bending and stretching states, as shown in Fig. 4b, c. The detailed demonstration of the proposed device flexibility can be found in supplementary Movie S1.

### Convolutional neural network simulation

The proposed device was operated with neuromorphic functionality, and the working data were obtained. To evaluate the proposed device, the simulation parameters for a convolutional neural network (CNN) can be easily obtained from these data^47^. Using these parameters for CNN simulation, CIFAR-10 recognition data were used, in which the input consists of 32 × 32 × 3. The first and second convolutional layers consist of 128 convolutional kernels of size 3 × 3 and subsampling data with size 2 × 2 after using two convolutional layers. The third and fourth convolutional layers consist of 256 convolutional kernels of size 3 × 3. Again, we subsample data with size 2 × 2 after using two convolutional layers. The fifth and sixth convolutional layers consist of 512 convolutional kernels of size 3 × 3. The feature map was connected to 2 fully connected layers for classification. The output layer is composed of 10 neurons for classification. In summary, 6 convolutional layers are used to extract features, and the last 2 fully connected layers are used to classify features^47^, as shown in Fig. 4d. The maximum conductance and minimum conductance of the device are 1.1400e−05 and 1.6385e−08, respectively, with an off/on ratio of ~5.4474, and nonlinearity for positive and negative pulse data is 2.23 and 2.81, respectively. As shown in Fig. 4e, the accuracy of the neural network is 84% and converges to 83% in epoch 20. A CNN is designed as shown in Fig. 4d, using 6 convolutional layers and 2 fully connected layers. The input image is CIFAR-10 data that have 10 classes^47^. For using CNN, our pursuit device array method is a parallel-read-out analog eNVM-based pseudo-crossbar, and details are discussed in Supplementary Figs. S1 and S2. The batch size is 200, and the epoch is 100. Based on these data, we trained kernels and synapses in all layers to minimize the error between the real output and predicted output^47^. For comparison of a real case, we also simulated the ideal device using a linear function. As shown in Fig. 4e, the ideal device presents a maximum accuracy of ~84.23% and converges to 84% in epoch 20. The hardware implementation of the CNN is given in Supplementary Figs. S1 and S2. The device shows stable cycle-to-cycle endurance repeatability, which is one of the most important parameters for on-chip training, as shown in Fig. 4f.

### Spike time-dependent plasticity

The spike time-dependent plasticity (STDP) rule helps to illustrate the practicability of the neuromorphic device for biological synapses^48^. We designed the pulse train, which consists of different amplitudes (+1.2, −1.2, −1, −0.8, −0.6, −0.3, and −0.1), to perform antisymmetric Hebbian as given in Fig. 4g, and a pulse scheme (−1.2, 1.2, 1, 0.8, 0.6, 0.3, and 0.1) was used to perform antisymmetric anti-Hebbian as given in Fig. 4h, where the pulse width is 600 µs and pulse off internal is 1 ms. The pre- and postsynaptic pulses combined to form a resulting signal more than the threshold voltage, which results in device conductance weakening or strengthening with the respective relative time interval Δ*t*, where the device conductance (Δ*W*) modulation is a function of the time interval (Δ*t*) between pre- and postsynaptic spiking. During Δ*t* > 0, prespike proceeds postspike, which results in potentiation, and conversely, for Δ*t* < 0, synaptic weight decreases (depression) due to postspike proceeds prespike^49,50^. The asymmetric Hebbian rule and the antisymmetric anti-Hebbian rule were performed using a flexible discrete channel resistive memory device, showing conductance change as a function of time interval Δ*t* between the pre- and postspiking pulse^51^. The STDP behavior of discrete channel resistive memory devices using different time intervals shows similar behavior as biological synapses^51^.

## Conclusion

In summary, we have addressed the key challenges in the use of soft and flexible multistate resistive switching in discrete channel device as a synapse by introducing a viscous IL BMIM FeCl_4_ and H_2_O for mimicking various shapes for the artificial intelligent neuromorphic system. The working mechanism was based on the ion 11 concentration polarization within the channel, which can be modulated through the electrical input signal. In this way, memorable conductance could be tuned by changing pulse width, frequency, and pulse amplitude. The SRDP and STDP behavior shows high stability to perform electronic synapses. The analog weight change behavior demonstrated a stable endurance performance with our discrete channel memristor. In the flexibility test, highly stable performance was archived under mechanical deformation. The proposed discrete channel synapse operating performance was evaluated using CIFAR-10 image recognition for system-level CNN simulation with an accuracy of 84%. We are sure that the paper gives insight for highly stable neuromorphic resistive memory device for wearable electronic systems.

## Materials and methods

### Device fabrication

FeCl_3_ and BMIM Cl were purchased from Sigma-Aldrich. Two grams of BMIM Cl was dried under vacuum in a round bottom flask placed in an oil bath at 120 °C. FeCl_3_ (1.86 g) was dried at 120 °C, and added to a flask containing BMIM Cl. The solution was placed again in an oil bath containing BMIM Cl and FeCl_3_ with a molar ratio of 1:1 and stirred under vacuum for 24 h. PDMS was purchased from Dow Corning. The curing agent and PDMS were mixed in 1:10, and the mold was prepared by placing a thin wire with a thickness of 1.5 mm and cured at 80 °C for 4 h, as shown in Fig. 1b. The length and width of the PDMS mold are 15 and 5 mm, respectively, as shown in Fig. 1b. A PDMS channel with a hole size of 1.5 mm was used to hold the IL, as shown in Fig. 1c. In the final step, IL BMIM FeCl_4_ and H_2_O were filled in a PDMS mold using a syringe with a volume ratio of 1:1 and two electrodes as anode and cathode used as contacts, and the liquid stopper was used on both sides to prevent the leakage of IL, as shown in Fig. 1d. The distance between both electrodes was kept at 0.5 mm to perform device characterization, as shown in Fig. 1e.

### Device characterization

The neuromorphic device was analyzed with the KEYSIGHT B2902A source measuring unit. The XPS spectrum was measured by a PHI 5600 (Physical Electronics) with an Al X-ray monochromator, which uses photoelectrons excited by X-ray emission for surface characterization up to a depth of 2–5 nm. FTIR was performed using a Bruker I.F.S. The Raman spectrum was measured by a HORIBA LabRAM HR confocal spectrometer equipped with an 800-nm-long monochromator. The He-Cd laser was shined on the surface of the sample with an excitation wavelength of 325 nm.

## Supplementary information

Supplementary File

Supplementary Movie S1
