# Supplementary material for: Ionic liquid multistate resistive switching characteristics in two terminal soft and flexible discrete channels for neuromorphic computing
Source: Microsyst Nanoeng. 2022 May 26;8:56. doi: 10.1038/s41378-022-00390-2 (PMC9135683; doi:10.1038/s41378-022-00390-2)
Supplement: Supplementary file 1 — Supplementary File [file 41378_2022_390_MOESM1_ESM.docx]

**Supporting information:**

**Ionic liquid multistate resistive switching characteristics in two terminal soft and flexible discrete channels for neuromorphic computing**

Muhammad Umair Khan^1,2,3^, Jungmin Kim^1^, Mahesh Y. Chougale^1^, Chaudhry Muhammad Furqan^4,5^,

Qazi Muhammad Saqib^1^, Rayyan Ali Shaukat^1^, Nobuhiko P. Kobayashi^6^, Baker Mohammad^2,3^,

Jinho Bae^1^* and Hoi-Sing Kwok^4,5^

^1^Department of Ocean System Engineering, Jeju National University, 102 Jejudaehakro, Jeju 63243, Republic of Korea.

^2^Department of Electrical Engineering and Computer Science, Khalifa University, Abu Dhabi127788, UAE

^3^System on Chip Center, Khalifa University, Abu Dhabi 127788, UAE

^4^Department of Electronic and Computer Engineering, The Hong Kong University of Science and Technology, Clear Water Bay, Kowloon, Hong Kong

^5^State Key Laboratory on Advanced Displays and Optoelectronics Technologies, The Hong Kong University of Science and Technology, Clear Water Bay, Kowloon, Hong Kong

^6^Baskin School of Engineering, University of California Santa Cruz, 1156 High Street, Santa Cruz, CA, 95064, USA

^*^E-mail: [baejh@jejunu.ac.kr](mailto:baejh@jejunu.ac.kr)

**CNN simulation architecture used for hardware implementation of neural network:**

For simulation, CIFAR-10 image is used as input data, which consists of 32×32×3. Final outputs of CNN with this dataset consist of 10 unit, in which classes with label value are converted into one hot vector. For error update, chain rule used to compare the truth label one hot vector and simulation output[^1^](#_ENREF_1). Our goal to use CNN simulation with memristor array. Device’s conductance is used as weight. we explained the simulation support following method to operate CNN in memristor array device. Fig. S1 shows the conventional mapping for convolution layer in which, kernel is flattened to long columns. And each kernels slide input data to element-wise multiplication with stride, and partial sum to extract output. Also, some input data will reuse for computation. In this way, first output feature map (OFM) element is from the sum of dot-product with the first kernel. The second OFM element is from the sum of dot-product with the second kernel. Therefore, we can get first element every OFM from all kernels. This process will be executed until last kernel and as a result, we can get output feature maps[^2^](#_ENREF_2) as shown in Fig. 3d of main manuscript.


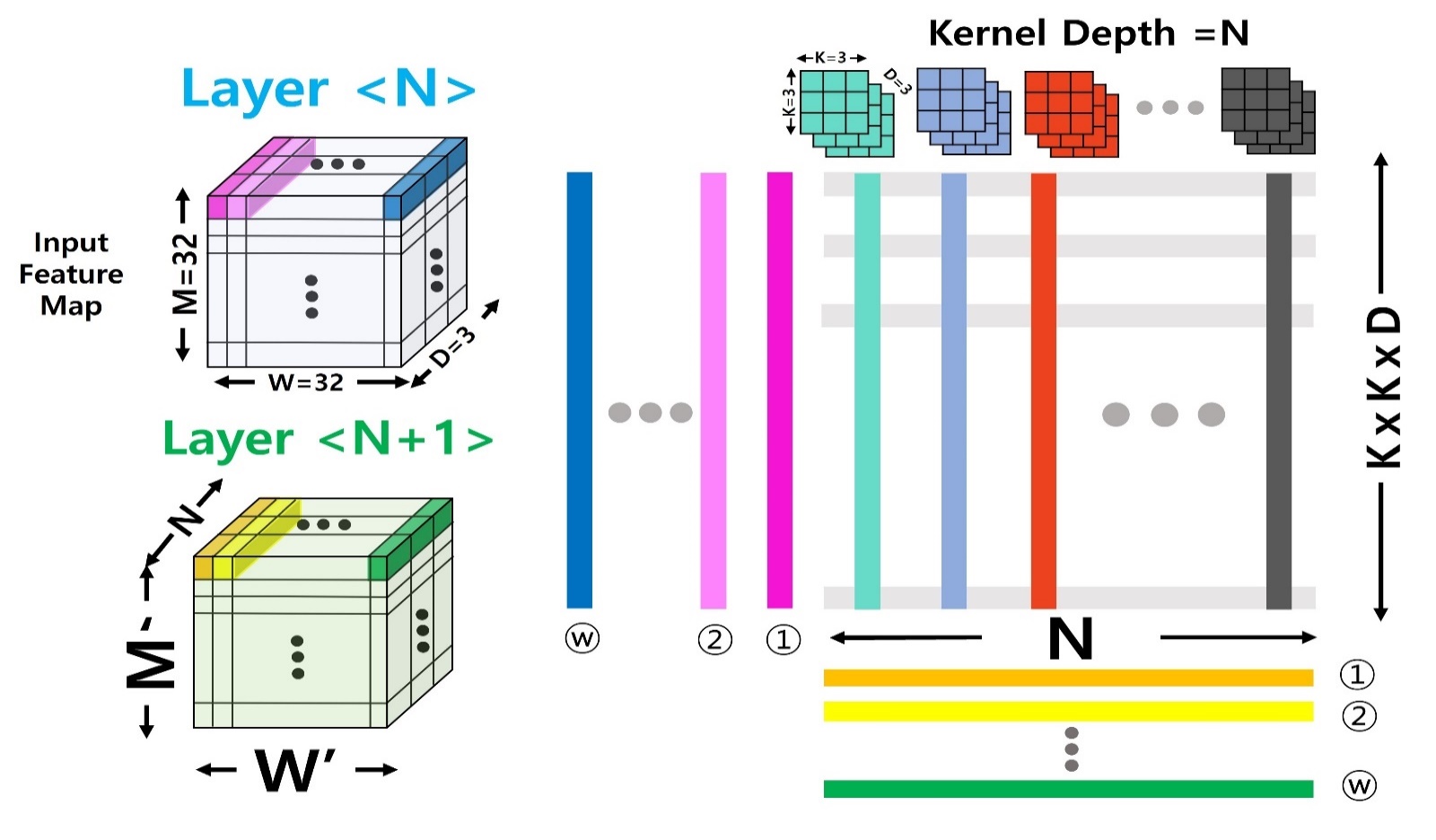


**Fig. S1.** The example of processing convolution with input and weight data.

Further the convolution neural network (CNN) can be implemented in a hardware manner as details are provided as follows: Further the convolution neural network (CNN) can be implemented in a hardware manner as given in Fig. S2 showing the computing operation architecture based on analog eNVM 1T1R synaptic array with sequential read out. Also, this figure shows how to treat cell array. The Word line (WL) can be considered as a switch, which controls the transistor. The source line (SL) is connected transistor source and bit line (BL) is connected the top electrode of eNVM cell. Bottom electrode is connected with the drain of the transistor. In this method, this array can’t perform parallel weight sum. So, BL is horizontal to WL as shown in Fig.S2. The BL receives input voltage, which results SL can read out weighted sum current. Switch matrix is connected to all BL and transmission gate control signals are stored in registers. During sum operation, input signal is loaded, and BLs connected to input voltage or ground. The role of WL/BL decoder make all transistors transparent for weighted sum. Multiplexer (MUX) is connected column line in the synaptic array. Mux shares the read periphery circuits with column line in memristor array. Resistive memory device uses as embedded nonvolatile memory (eNVM). When input comes in eNVM, output will be current. These eNVM arrays perform CNN algorithm as shown in Fig.S1. Adder and register are integrated to all weight sums. At the bottom of synaptic core the adder and shift register shifts and add all weighted sum result at each input vector bit cycle to get the final weighted sum[^3^](#_ENREF_3).


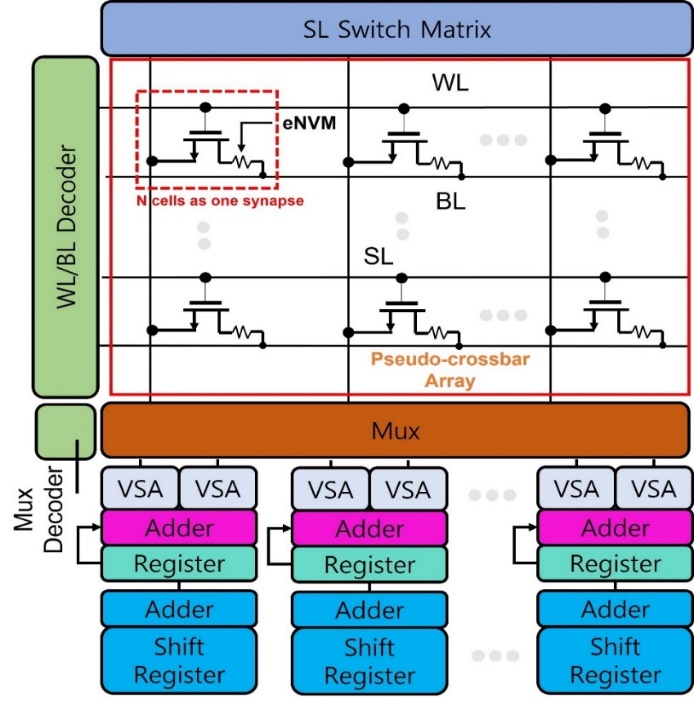


**Fig. S2.** Analog eNVM ITR synaptic array with sequential read out.

In neural network, each neuron layer is connected to every neuron of next layer, each connections represents weight. The weight can be updated using memristor crossbar array to make a neural network. So, weight can be replaced by conductance G_i_. Therefore, vector matrix multiplication can be operated this crossbar array by Ohm’s law and Kirchhoff’s law efficiently. The matrix function can be expressed as follow:

$\left[ \begin{matrix} I_{1} \\ \vdots\\ I_{N} \end{matrix} \right]=\left[ \begin{matrix} G_{11} & \cdots& G_{1N} \\ \vdots& \ddots& \vdots\\ G_{N1} & \cdots& G_{NN} \end{matrix} \right] \left[ \begin{matrix} V_{1} \\ \vdots\\ V_{N} \end{matrix} \right] (1$)

Where $V_{n}$ is input voltages, $I_{n}$ is output current and $G_{ij}$ is conductance. Therefore, output $I_{n}$ can be collected from each bit line. In detail process for training, it has two main steps, which is the feed forward and back propagation process. The role of feed forward process is utilized to obtain error between the output result and true value. In back propagation process, the weights are updated based on feed forward result. Update through back propagation process is repeated iteration process until error is eliminated. Especially, if update for back propagation process, the nonlinear weight is used according to conductance of memristor.

**Comparison of liquid neuromorphic resistive memory devices.**

| **No** | **Liquid neuromorphic resistive memory Device structure**  **& Device flexibility** | **Device operating voltage, endurance & retension** | **Spiking voltage and number of pulses for potentiation & depression &**  **Spiking pulse width** | **Weight ratio** | **Spiking stability** | **Simulation based on neural network or convolutional neural network** | **SRDP** | **Ref:** |
| --- | --- | --- | --- | --- | --- | --- | --- | --- |
| **1** | Ag/H_2_O/Au  (glass substrate)  (not flexible) | Bipolar resistive swiching  ±0.2 V  (200 cycles endurance) | + 0.2 V and -0.2 V  (400 pulses for Potentiation and Depresssion)  (0.2 sec pulse width) | ~ 4.5 | - | - | - | [**^4^**](#_ENREF_4) |
| **2** | Ag/AgNO_3_/Probe_Tip  (Silicon Substarte)  (not flexible) | Bipolar resistive swiching  - 0.15 V to + 0.3 V  (100 cycle to cycle endurance) (retention 1000 sec) | - 60 mV and + 90 mV  (10 pulses for Potentiation & depression)  (0.1 sec pulse width) | ~ 5 | - | - | - | [^5^](#_ENREF_5) |
| **3** | Cu/Ag@AgCl/Cu  (PDMS substrate) (Flexible) | Multistate resistive swiching  ± 1.5 V  (100 cycle to cycles endurance) | +1.3 V and -1.3 V  (100 pulses for potentiation and depression)  (1 ms pulse width) | ~ 1.5 | 400 pulses | Convolutional neural network accuracy 85% | - | [**^6^**](#_ENREF_6) |
| **4** | Au/Trypsin/FTO  (Glass substarte)  (not flexible) | Multistate resistive swiching  ±1V to ±10 V  (1 V voltage step for each sweep) | + 4 V and – 4 V  (50 pulses for Potentiation and depression)  (10 ms pulse width) | ~ 14 | 500 pulses | - | yes | [**^7^**](#_ENREF_7) |
| **5** | Ag/AgCl/KCl/BMIM PF_6_/AG/AgCl  (PDMS substrate)  (Flexiblity test not performedk) | Bipolar resistive swiching  ± 20 V  (50 cycle to cycle endurance) | + 4 V and - 10 V, 1V(read)  (80 pulses for Potentaion and Depression**)**  (0.5 sec pulse width) | ~ 2 | 1000 pulses | Convolutional neural network accuracy 97% | - | [**^8^**](#_ENREF_8) |
| **6** | **Cu/BMIM FeCl_4_ : H_2_O/Cu**  **(PDMS substrate: highly Flexible and robust)**  **(Flexible)** | **Multistate resistive swiching**  **± 1.5 V** | **+1.5 V and -1.5 V**  **(30 pulses for potentiaon and depression)**  **(1ms pulse width)** | **~ 4** | **180 Pulses** | **Convolutional neural network accuracy 84%** | **yes** | **This work** |

**Table. S1.** Comparison of ionic liquid and hydrogel neuromorphic resistive memory devices.

**Reference**

1 Liu, S. & Deng, W. in *2015 3rd IAPR Asian Conference on Pattern Recognition (ACPR).* 730-734.

2 Peng, X., Liu, R. & Yu, S. in *2019 IEEE International Symposium on Circuits and Systems (ISCAS).* 1-5.

3 Chen, P., Peng, X. & Yu, S. NeuroSim: A Circuit-Level Macro Model for Benchmarking Neuro-Inspired Architectures in Online Learning. *IEEE Transactions on Computer-Aided Design of Integrated Circuits and Systems* **37**, 3067-3080, doi:10.1109/TCAD.2018.2789723 (2018).

4 Ananthakrishnan, A., Du, X. & Allen, M. G. Water-based resistive switches for neuromorphic long-range connections. *Journal of Physics D: Applied Physics* **54**, 225104, doi:10.1088/1361-6463/abead7 (2021).

5 Kim, D. & Lee, J.-S. Liquid-based memory and artificial synapse. *Nanoscale* **11**, 9726-9732, doi:10.1039/C9NR02767J (2019).

6 Khan, M. U. *et al.* Soft and flexible: core-shell ionic liquid resistive memory for electronic synapses. *Microsystems & Nanoengineering* **7**, 78, doi:10.1038/s41378-021-00305-7 (2021).

7 Desai, T. R. *et al.* Synaptic learning functionalities of inverse biomemristive device based on trypsin for artificial intelligence application. *Journal of Materials Research and Technology* **11**, 1100-1110, doi:https://doi.org/10.1016/j.jmrt.2021.01.108 (2021).

8 Zhang, P. *et al.* Nanochannel-Based Transport in an Interfacial Memristor Can Emulate the Analog Weight Modulation of Synapses. *Nano Letters* **19**, 4279-4286, doi:10.1021/acs.nanolett.9b00525 (2019).
